# Clinical predictors of surgical intervention for gastrointestinal magnetic foreign bodies in children

**DOI:** 10.1186/s12887-023-04125-8

**Published:** 2023-06-24

**Authors:** Rui Yun Zhang, Peng Cai, Ting Ting Zhang, Jie Zhu, Jian Lei Chen, Hao Wei Zhao, Yu Liang Jiang, Qi Wang, Meng Lei Zhu, Xiao Gang Zhou, Xian Lan Xiang, Fei Long Hu, Zhi Cheng Gu, Zhen Wei Zhu

**Affiliations:** Department of Pediatric Surgery, Children’s Hospital of Soochow University, Suzhou, 215127 Jiangsu Province China

**Keywords:** Magnetic foreign body, Timing of surgery, Complications

## Abstract

**Background/aims:**

To investigate the clinical situation, treatment methods, and clinical predictors of surgical intervention in children with magnetic foreign bodies in the digestive tract.

**Materials and methods:**

From January 2019 to June 2022, we retrospectively analyzed the clinical data of 72 children who ingested magnetic foreign bodies inadvertently in our hospital, including their general information, admissions, clinical manifestations, and treatment methods, as well as pertinent literature and statistical data. Following software processing, univariate and multivariate logistic regression analyses were conducted to determine the independent risk factors of this study.

**Results:**

In this study, 16 patients (22.2%) were discharged smoothly following conservative treatment and 19 patients (26.4%) were cured by gastroscopy. The remaining 37 patients (51.4%) were underwent surgery, in which 26 cases developed gastrointestinal perforation. There were statistical differences between surgery group and non- surgery group in the days of eating by mistake, clinical manifestations (nausea and vomiting, intermittent abdominal pain, abdominal muscle tension) and movement trajectory by every 24-h radiograph (*P* < 0.01). Logistic regression analysis showed that intermittent abdominal pain and abdominal muscle tension were independent risk factors for surgical treatment.

**Conclusion:**

Magnetic foreign bodies seriously endanger children’s health. This study offers a single-center basis for the choice of surgical opportunity for intestinal obstruction or perforation caused by magnetic foreign bodies. Clinicians need immediate surgical intervention if the child shows symptoms of abdominal pain or abdominal tension.

## Introduction

One of the significant causes of accidental injury in children is ingesting foreign bodies through the digestive tract, primarily because children have a limited ability to perceive things and an intense curiosity [1]. For treating foreign bodies in the digestive tract, conservative self-discharge, gastroscopic removal, and surgical procedures are currently the most common options. According to pertinent statistics, over 80% of cases of oral ingestion of foreign bodies are treated conservatively, and only 1% of children require surgical intervention [2]. Common types of foreign surgical bodies include magnetic foreign bodies and button batteries. Different types, quantities, and shapes of foreign bodies also result in distinct complications. Suppose a child accidentally consumes a large quantity or multiple batches. In that case, the magnetic substances may attract each other in the digestive tract, leading to hypoxia-induced extrusion of the gastrointestinal tract wall, intestinal necrosis, perforation, obstruction, or formation of a fistula [3]. In the absence of timely surgical intervention, volvulus can develop; in severe cases, toxic shock could occur, which may be life-threatening. Due to the slow development and hidden nature of magnetic foreign body disease in the digestive tract, early diagnosis, selection of appropriate treatment methods, and determining the timing of surgery are crucial. Current literature reports and diagnosis and treatment guidelines do not straightforwardly explain the selection of the timing of magnetic foreign body surgery. Numerous factors are associated with the surgical intervention of children who ingest magnetic foreign bodies by accident, so this study retrospectively analyzed the children’s clinical data and other factors. Through pertinent data, we can predict the relevant factors of surgical intervention and provide a foundation for clinical diagnosis and treatment.

## Materials and methods

### Clinical data

Seventy-two children admitted to our hospital between January 2019 and June 2022 due to the accidental ingestion of magnetic foreign bodies were chosen as the research subjects. It was reviewed and approved by the Children’s Hospital Affiliated with Soochow University’s Ethics Committee (NO.2022CS153). According to the treatment method, all children were divided into surgery group (laparoscopic, Laparotomy) and non-surgery groups (conservative, gastroscopic removal) and the admission situation of the two groups was analyzed. The relevant data were analyzed and presented. The study was approved by the institutional review board of Children’s Hospital of Soochow University. The authors have identified the institutional and/or licensing committee approving the experiments, including any relevant details; confirming that all experiments were performed in accordance with following relevant guidelines and regulations. Informed consent was waived by the institutional board of Children’s Hospital of Soochow University as a retrospective study is performed.

### Inclusion and exclusion criteria

Inclusion criteria: there must be a clear history of ingestion of magnetic foreign bodies, or endoscopy, imaging, and surgery must indicate the presence of magnetic foreign bodies.

Exclusion criteria: the foreign body in the digestive tract is non-magnetic, and the patient cannot cooperate due to other issues.

### Observation index

The relevant clinical data of all children who accidentally ingested magnetic foreign bodies include age, gender, time from onset to consultation, primary symptoms, type of accidental ingestion, number of accidental ingestions, supplementary examination, treatment method, complications, and prognosis.

### Statistical analysis

By examining the inpatient medical records, a database was established. Excel was utilized to establish a database. Two individuals entered data, which a third person reviewed. The statistical analysis was conducted with SPSS 17.0. Various statistical methods were used to analyze the data. Normally distributed data in measurement data were expressed as mean ± standard deviation (x ± s), and comparisons between groups were made using an independent samples t-test. Skewed distribution data were expressed as median numbers and interquartile ranges, and comparisons between groups were made using the Mann-Whitney U test. Enumeration data were expressed as n (%), and χ2 test or Fisher’s test was utilized for group comparison. All statistical tests were two-sided, and *P* values < 0.05 were considered statistically significant. Among the relevant risk factors compiled are univariate logistic regression analysis. To exclude confounders and include all relevant risk factors, all factors with* P* < 0.1 were included in multiple logistic regression analysis. This study’s final statistically significant (*P* < 0.05) factor was an independent risk factor.

## Results

### Admission and treatment of children who ingested magnetic foreign bodies

**Fig. 1:**
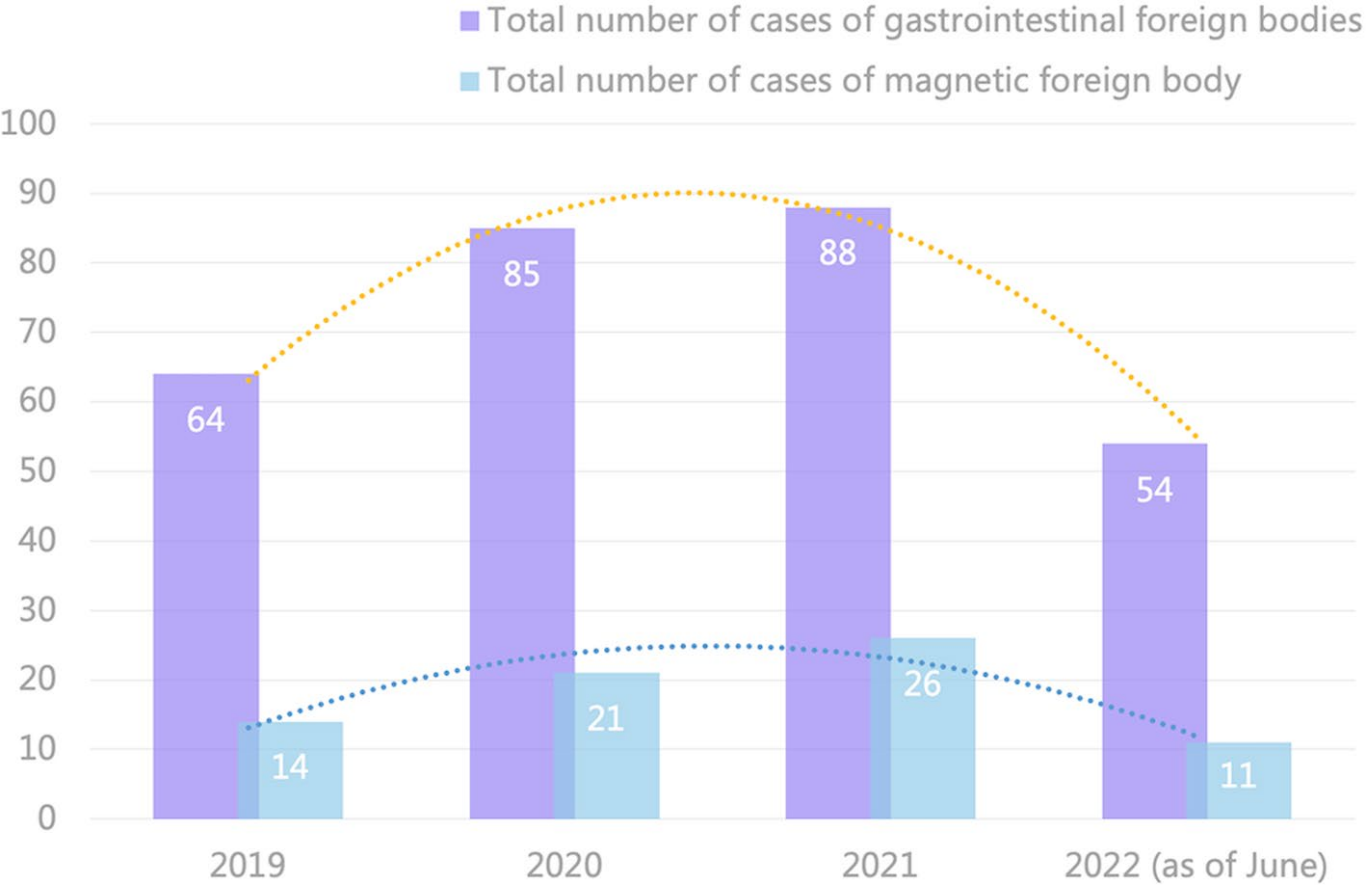
The hospital’s income and expenditure related to foreign bodies in the digestive tract and the proportion of magnetic foreign bodies from January 2019 to June 2022

The collected case information was categorized and summarized according to admission years and treatment methods. See Table 1 for details. In our hospital, the incidence and cost of gastrointestinal foreign bodies in hospitalized children ranged from 54 to 88 cases per year. In the first half of 2022, there were 54 cases of foreign bodies in the digestive tract, of which 11 (20.4%) were magnetic foreign bodies. The specific data are depicted in Fig. 1.

**Table 1:** Admission and treatment of children who mistakenly ingested magnetic foreign bodies

Year	Total number of cases (cases)	Conservative treatment (cases)	Gastroscopy removal (cases)	Surgical treatment (cases)	Proportion of surgical interventions
2019	14	2	4	8	57.14%
2020	21	4	4	13	61.90%
2021	26	8	7	11	42.31%
2022 (as of June)	11	4	2	5	45.45%
Total	72	18	17	37	51.39%

### Magnetic foreign bodies in the gastrointestinal tracts of children: prevalence and clinical manifestations

**Fig. 2:**
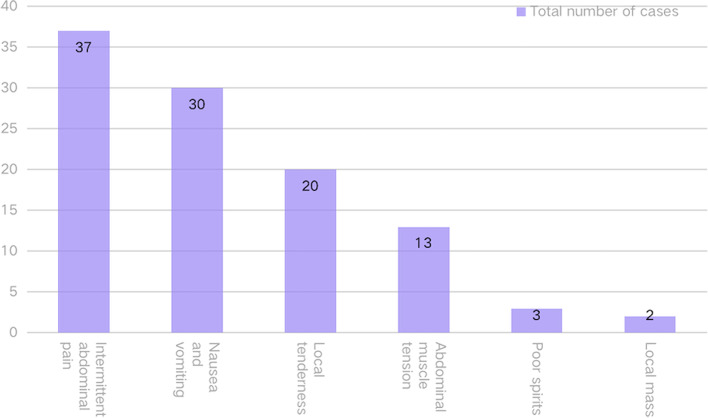
Clinical manifestations of children who mistakenly ingested magnetic foreign bodies

**Fig. 3:**
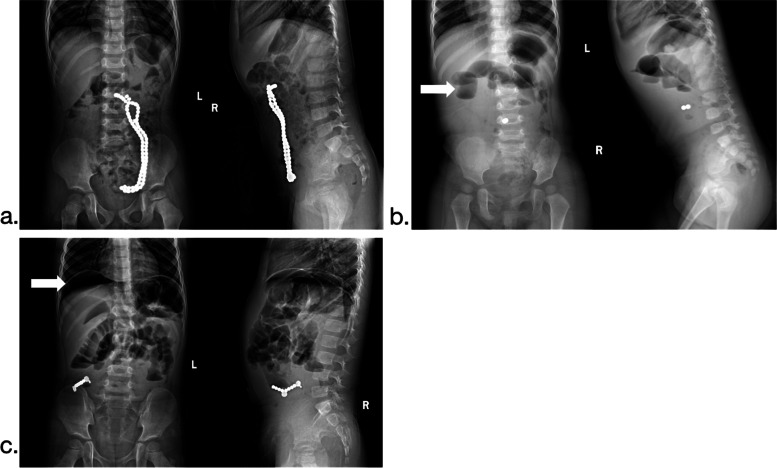
**a** Numerous magnetic foreign bodies, as determined by a multi-angle chest and abdomen X-ray. **b** Intestinal obstruction caused by magnetic foreign bodies with dilated proximal small bowel loops (white arrow). **c** Gastrointestinal perforation caused by magnetic foreign bodies, with sub-diaphragm free gas (white arrow)

In the cases, vomiting, persistent abdominal pain, diarrhea, and parental findings or self-reported ingestion of magnetic beads were the most common reasons for medical treatment; the most common clinical manifestations were nausea and vomiting (42.62%), persistent abdominal pain (57.38%), abdominal muscle tension (13.11%), local mass (3.28%), local tenderness (27.87%), and poor spirit (3.28%). Refer to Fig. 2. To further confirm the diagnosis, the upright abdominal X-ray is frequently utilized in the clinic as the preferred supplementary examination. This study’s abdominal X-ray examination can detect foreign bodies in the gastrointestinal tract. Figure 3 demonstrates the typical X-ray findings.

### A comparison of treatment strategies for children who ingested magnetic foreign bodies

Of the 72 children in this study, 9 (12.5%) were under 1 year old, 26 (36.11%) were between1 to 3 years, and 55 (76.4%) were between 3 to 6 years, with a gender ratio of 3.69:1 (Male:Female). The number of accidental ingestions ranged from 2 to 95, with the most extended duration exceeding 1 month. In the cases included in this article, magnetic foreign bodies in the digestive tract are primarily treated with observation, gastroscope removal, and surgery. There were 37 surgical intervention cases (51.39%) and 35 non-surgical intervention cases (48.61%). Age, gender, ingested number by mistake, local tenderness on physical examination, and mass did not differ between the two groups. As for statistical significance, on the contrary, there were statistically significant differences between the groups regarding the time of ingesting by mistake, clinical manifestations (e.g., nausea, vomiting, persistent abdominal pain, abdominal muscle tension) and 24-h radiograph movement (*P* < 0.05). The information is presented in Table 2.

**Table 2:** Comparison of clinical data between the surgical and non-surgical groups

	Surgery group (*n* = 39)	Non-surgical group (*n* = 33)	Z/χ^2^	*P*
Age/Year^b^	4.00 (3.00, 5.00)	5.00 (3.00, 8.50)	-1.33	0.184
Gender				
Male:Female^a^	3.6:1	4.6:1	0.13	0.910
Accidental ingestion time/day^b^	2.00 (1.00, 4.00)	0.72 (0.23, 1.63)	-3.55	0.001♦
Accidental ingestion number/capsule^b^	7.00 (5.00, 10.75)	5.00 (3.00, 9.00)	-1.515	0.130
Clinical manifestations				
Nausea^a^	15	7	7.193	0.007♦
Vomiting^a^	15	7	6.696	0.010♦
Intermittent abdominal pain^a^	28	8	6.771	0.001♦
Abdominal muscle tension^a^	15	0	6.436	0.011♦
Local mass^a^	1	1	0	1.000
Local tenderness^a^	13	5	3.349	0.067
Movement trajectory by every 24-h radiograph^a^	0	17	15.815	0.001♦

Items with “^a^” used chi-square test for data analysis, and data are described above by frequency; items with “^b^” used Mann-Whitney U test, and data are described above by median (interquartile range). Finally, items with “♦” refer to *P* < 0.05, which denotes statistical significance

### Analysis of factors related to surgical intervention of magnetic foreign bodies in children’s digestive tract

Initially, univariate analysis was performed. Magnetic foreign bodies in the digestive tract are associated with accidental eating time, clinical manifestations (nausea, vomiting, persistent abdominal pain, abdominal muscle tension), and movement trajectory by every 24-h radiograph related to surgical procedures. Therefore, significant univariate factors were included in the multivariate logistic regression analysis, excluding confounding variables. In children with magnetic foreign bodies in the digestive tract, abdominal muscle tension and intermittent abdominal pain are independent risk factors for surgical intervention. See Table 3 for details.

**Table 3:** Logistic regression analysis of factors related to surgical intervention for magnetic foreign bodies in the gastrointestinal tract

	Univariate regression analysis			Multivariate regression analysis		
	OR	95%CI	*P*	OR	95%CI	*P*
Accidental ingestion time/day	0.890	0.764–1.035	0.130			
Movement trajectory by every 24-h radiograph	1.350	0.004–0.299	0.002♦			
Clinical manifestations						
Nausea	4.952	1.239–19.789	0.024♦			
Vomiting	5.000	1.606–15.566	0.005♦			
Intermittent abdominal pain	6.667	1.816–16.810	0.003♦	1.177	0.834–1.918	0.039♦
Abdominal muscle tension	0.090	0.018–0.040	0.003♦	18.417	2.115–20.976	0.008♦

Items with “♦” refer to *P* < 0.05, which is statistically significant

### Digestive tract perforation resulting from magnetic foreign bodies in the gastrointestinal tract

For 26 (42.6%) of the 72 children with magnetic foreign bodies in the gastrointestinal tract, gastrointestinal perforation and the formation of an internal fistula were present. Most perforations occur in the ileum, colon, or stomach. The information is provided in the table below. Among the 39 children with magnetic foreign bodies treated by surgery, 26 cases showed gastrointestinal perforation (66.7%), including magnetic beads (22, 84.62%) and other magnetic bodies (4, 15.38%). The numbers of perforation ranged from one to four sites, and there was only one perforation in most cases (14, 53.85%) (Table 4). The perforation sites were very diverse and unexpected, including jejunum or ileum (15,57.69%), stomach—jejunum/ileum (3,11.54%), stomach—colon (1,3.85%), jejunum/ileum—colon (6,23.08%) and others (1,3.85%).

**Table 4:** Clinical data of some children with perforation caused by magnetic foreign bodies in the digestive tract

	Number of cases (*n* = 26)	Proportion(%)
Foreign body type		
Magnetic bead	22	84.62%
Other magnetic foreign bodies	4	15.38%
Perforation number		
1	14	53.85%
2	7	26.92%
More and 3	5	19.23%
Perforation site		
Stomach—jejunum/ileum	3	11.54%
Stomach—colon	1	3.85%
Only jejunum/ileum	15	57.69%
Jejunum/ileum—colon	6	23.08%
Others^a^	1	3.85%

^a^The perforation position was in Meckel’s diverticulum

## Discussion

Foreign bodies in the gastrointestinal tract remain as the common causes of pediatric surgical emergencies and are especially prevalent in preschoolers. As our data show, the age of most children were under 6 years, and cases were more common in boys [4], it is consistent with the results of the 2015 U.S. Epidemiological Survey on Foreign Bodies in the Digestive Tracts of Children regarding the age and gender ratios [5].

Magnetic toys consist primarily of magnetic beads, rods, and cubes, with magnetic beads being the most prevalent. They have vivid colors, high magnetic energy products, and coercive force, demonstrating their potency and the danger they pose to children who accidentally ingest them [6]. The sale of magnetic toys has been strictly regulated in recent years due to societal pressure and the intervention of relevant departments [7]. According to the data analysis results of this single center in our hospital, the number of children with magnetic foreign bodies has not decreased significantly Therefore, the diagnosis, treatment, and prevention of magnetic foreign bodies in children’s digestive tracts are crucial.

### Clinical manifestations of accidental ingestion of magnetic foreign bodies in the digestive tract

Magnetic foreign bodies in the digestive tract of children are a relatively common and highly unique category of foreign bodies in the digestive tract that are primarily determined by their physical characteristics. Accidental ingestion of a single magnetic foreign body typically results in few clinical manifestations, and most are excreted naturally. However, it dramatically increases the likelihood that the gastrointestinal tract, mesentery, and other tissues will be compressed. Prolonged ischemia will cause necrosis, perforation, and fistulas in the gastrointestinal tract. In this group of children with magnetic foreign bodies in the digestive tract, the number of accidental ingestions ranged from 2 to 95, with the most extended duration exceeding 1 month. Consequently, the severity of the clinical manifestations varies. Common symptoms include intermittent abdominal pain, nausea and vomiting, abdominal distension, and diarrhea. Severe cases can rapidly develop life-threatening intestinal obstruction, peritonitis, and shock.

Previous research has demonstrated that magnetic beads attract one another and rarely cause abdominal leakage. The primary cause of the symptoms is intestinal obstruction. These children develop internal fistulas because of long-term perforation. Compression of the intestinal tube leads to intestinal root obstruction in a closed intestinal loop. Intestinal obstruction caused by magnetic foreign bodies is mainly manifested as vomiting, abdominal pain, abdominal distension and gastrointestinal type visible in the abdomen, whereas abdominal pain is mostly concentrated around the umbilicus or upper abdomen, and it is manifested as high obstruction on X-ray (Fig. 3b). The results of this study suggest that nausea, vomiting, abdominal pain, tenderness or rebound tenderness are closely related to surgical treatment. Therefore, it is suggested that once magnets in digestive tract is clinically diagnosed with the previous symptoms, emergency surgery should be considered, the more severe of obstruction, the higher priority of operation.

### Selection of examination methods

The most common clinical imaging examination is a thoraco-abdominal X-ray, which frequently reveals multiple high-density shadows distributed in a beaded or clumpy pattern. Multi-angle abdominal X-ray photography and CT scan can be used when the number of magnets are uncertain (Fig. 3a). Dynamic abdominal X-ray monitoring is an important indication of operation. Unfortunately, abdominal B-ultrasound is not specific for diagnosing magnetic foreign bodies in the digestive tract, resulting in a high rate of missed diagnoses. The type of perforation caused by magnetic foreign bodies in the digestive tract is uncommon, and it typically has a small diameter and is predominantly an internal fistula. Therefore, abdominal X-rays reveal less free gas under the diaphragm [8]. There were only 2 cases appeared subphrenic free gas (Fig. 3c).

### Diagnosis and treatment of ingestion of magnetic foreign bodies in digestive tract

A complete and comprehensive medical history is essential for diagnosing and treating this type of illness. Children who ingest magnetic foreign bodies inadvertently typically have a clear history of exposure to magnetic foreign bodies. Unfortunately, time is not adequately described. Consequently, the diagnosis of accidental ingestion of magnetic foreign bodies in the digestive tract is frequently based on clinical manifestations combined with imaging examinations, with abdominal X-ray being the most important (frontal and lateral view). It is intuitively reflected and provides practical assistance in selecting follow-up treatment options.

**Fig. 4:**
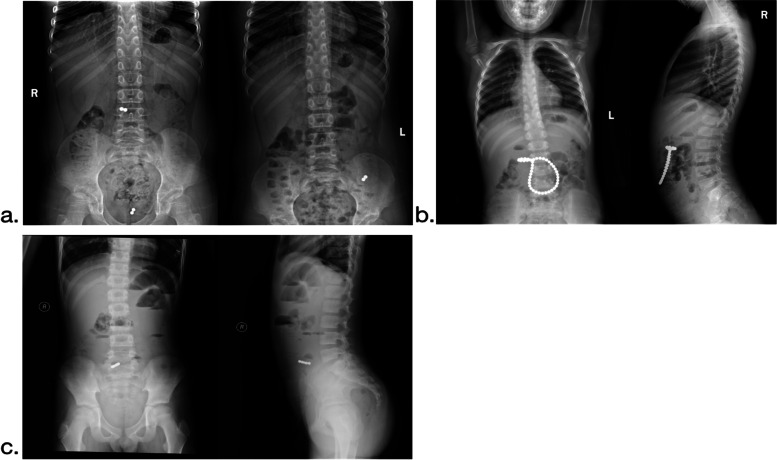
**a** 24-h dynamic radiographs for moving patients, and conservative treatment is performed. **b** The combined thoracic and abdominal radiograph confirms that the foreign body is in the duodenum, and the foreign body is extracted via gastroscopy. **c** During the operation, the combined chest-abdominal radiograph confirms the presence of magnetic foreign bodies causing intestinal obstruction. Meckel’s diverticulum is the location of the perforation in the digestive tract

Presently, the most frequently used clinical treatments for magnetic foreign bodies in the digestive tract are the self-discharge of foreign bodies, gastroscopy, and laparoscopic foreign body removal. Dynamic abdominal X-ray monitoring is the current “gold standard” for clinical guidance [8], which is consistent with our research. If the patient is not in critical condition, and the magnets are persistently moving appeared in X ray, conservative treatments are considered preferentially (Fig. 4a), most of these magntic beads can be discharged within 3–5 days. If the magnetic beads continuously move to the ileocecal valve and then are fixed, we could still observe another 3–5 dayss as well as laxative therapy. However, if the magnatic beads in the children’s radiographs are fixed combine with clinical manifestations, surgical treatment should be performed to avoid serious complications.

Foreign bodies can be removed under a gastroscope from children who have swallowed them by accident or if the foreign body is in the duodenum or above within 24 h. If the gastroscope cannot be removed entirely, the foreign body’s dynamic changes should be closely monitored, and surgery should be performed if it remains for an extended period [9]. As depicted in Fig. 4b, Continuous radiographs showed all the foreign bodies were located in stomach, we removed them successfully by gastroscopy. In this study, a gastroscopy was performed on 16 children, and the success rate was 68.75%, which has the benefits of a high success rate, minimal damage, and low cost.

**Fig. 5:**
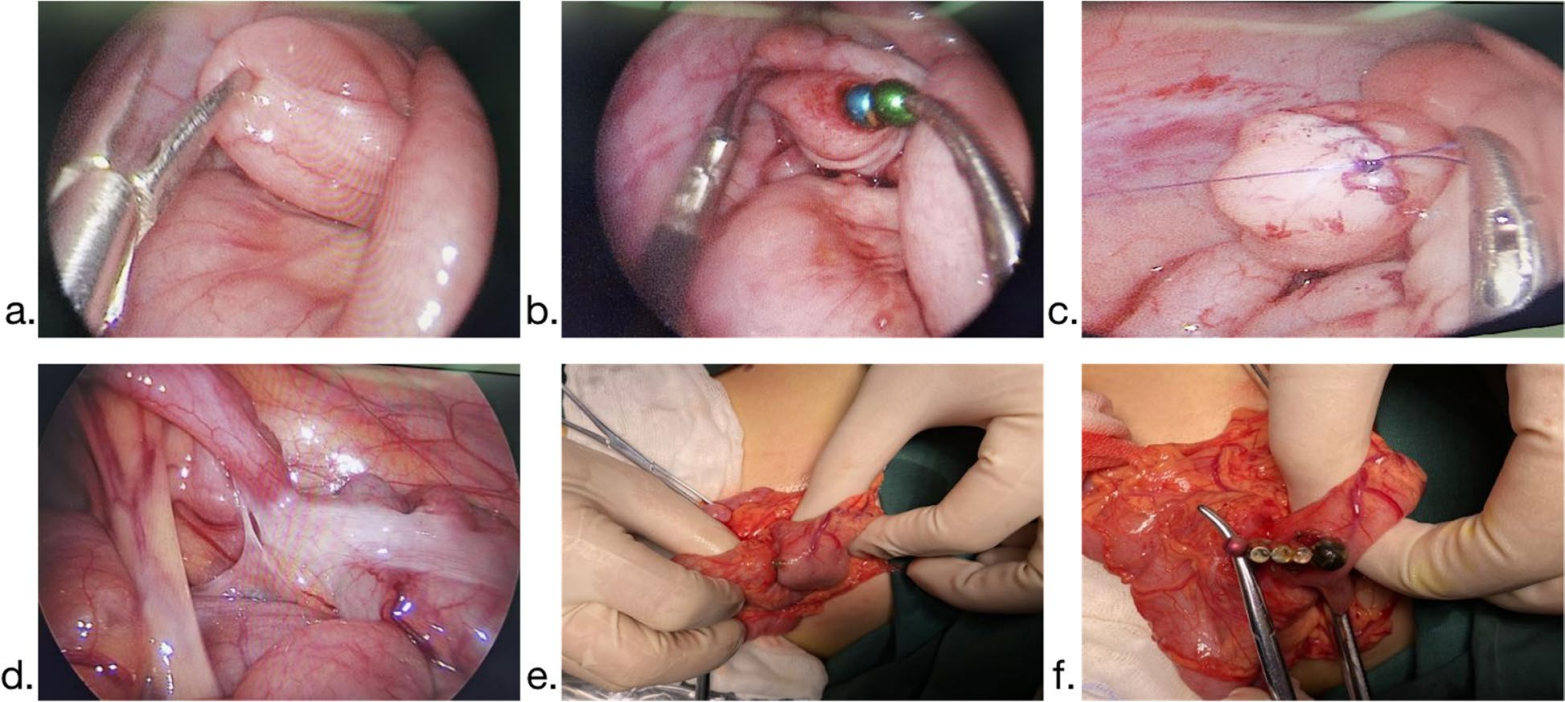
**a**, **b**, **c** Laparoscopic examination of magnetic foreign bodies with metal grasping forceps, followed by small bowel incision and removal of magnetic beads, and then suturing. **d**, **e**, **f** Laparoscopic exploration revealed obvious perforation and adhesion. The surgical incision was expanded at the umbilicus, the perforated part of the bowel was removed, alimentary tract foreign body removal was removed, intestinal adhesion was released, and intestinal perforation was eventually repaired

Gastroscopy combined with laparoscopy has great advantages for complicated magnetic bead cases, and this not only reduces risks for repeated anesthesia, but also accidentally finds out the foreign remaining in the esophagus. Immediate surgical treatment should be administered to patients who did not respond to gastroscopy removal, those with obvious signs of peritoneal irritation, and those with confirmed gastrointestinal magnetic foreign bodies and unstable vital signs. Some scholars believe that laparotomy should be the first choice for surgical intervention [10]. We agree that laparoscopic exploration should be the first choice for surgical intervention, primarily because it is not only a surgical method but also a visual inspection. Laparoscopic exploration can assist in diagnosing patients who cannot identify the location of the foreign body on an abdominal X-ray and who are unable to judge the necrosis and perforation [11] (Fig. 5a, b, c). In comparison to traditional laparotomy, laparoscopic surgery causes less damage. Simultaneously, it is possible to enlarge the umbilical incision and remove the free intestinal tube that is absorbed only by foreign bodies. The intestinal canal of an internal fistula can also be used to locate the abdominal wall incision more precisely, thereby preventing blind expansion of the incision. When there is apparent necrosis and perforation, or the endoscopic procedure is complex, it should be converted to laparotomy in time to ensure a smooth and smooth operation of the digestive tract (stomach to rectum) to prevent missed perforation sites (Fig. 5d, e, f). In this study, 21 instances of laparoscopic surgery were converted to open surgery, accounting for 65.6% of all surgical procedures. La laparoscopic exploration frequently reveals necrosis, perforation, or the formation of an internal fistula due to the absence of a clear opportunity for surgical intervention, which is the primary cause of its high incidence [11].

### Complications and prognosis of the ingestion of magnetic foreign bodies in the digestive tract

Complications caused by magnetic foreign bodies in the digestive tract of children frequently have a direct bearing on their treatment, hospital stay, hospital costs, and prognosis [12]. The principal complications include mucosal edema, erosion, intestinal necrosis, perforation, bleeding, and intestinal obstruction, with gastrointestinal perforation being the most prevalent. Digestive tract perforation accounted for in this study accounts for more than 70% of surgical treatment cases. Among them, patient No. 19 was found to have Meckel’s diverticulum during the operation. Upon perforation, he underwent “Meckel’s diverticulum excision, gastrointestinal foreign body removal, intestinal adhesion release, enteroenteric anastomosis, and abdominal drainage,” and the postoperative recovery was acceptable (Fig. 4c). Patient No.9 showed another complicated perforation locations, the magnetic beads penetrated through the descending duodenum as well as ascending colon mesentery and located around appendix. We then performed “digestive tract foreign body removal and intestinal perforation repair” subsequently. Patient No. 23 ingested magnetic beads and button batteries by accident. Due to the high corrosiveness of button batteries, the degree of intestinal adhesion during the operation was severe, and most of the intestinal tubes were necroticwe immediately resected the necrotic intestine and performed adhesive tape loosening and intestinal anastomosis. The child developed intestinal obstruction after the surgery but gradually improved with conservative treatment. According to the prognosis survey of all children in this study, most children have been completely cured, and there were two cases of postoperative intestinal obstruction [13, 14].

In conclusion, the damage caused by magnetic foreign bodies in children’s digestive tracts is highly severe, so prevention is frequently preferable to treatment. Therefore, we should urge the social level to actively promote education, discourage the use of magnetic toys, and remind relevant manufacturers to create visible signs. Awaken all parents and safeguard children from contact. Medical personnel must actively adopt a treatment plan for children who have ingested by accident, prioritizing the use of gastroscope removal or gastroscope combined with laparoscopy to improve the foreign body removal rate, reduce the incidence of complications, and ensure the safety of children’s lives [15, 16].

## Data Availability

The datasets used or analysed during the current study are available from the corresponding author on reasonable request.
